# Bone Marrow-Sparing Pelvic Radiotherapy: Clinical Evidence, Dosimetric Constraints, and Clinical Outcomes

**DOI:** 10.7759/cureus.101210

**Published:** 2026-01-10

**Authors:** Reyzane El Mjabber, Rim Alami, Asmaa Naim, Fadila Kouhen, Nabil Ismaili, Sanaa El Majjaoui, Zineb Dahbi

**Affiliations:** 1 Radiation Oncology, Mohammed VI University of Sciences and Health (UM6SS), Casablanca, MAR; 2 Radiation Oncology, Cheikh Khalifa International University Hospital, Casablanca, MAR; 3 Radiation Oncology, Mohammed VI University of Sciences and Health (UM6SS), Rabat, MAR; 4 Radiation Oncology, Mohammed VI International University Hospital, Rabat, MAR; 5 Medical Oncology, Mohammed VI University of Sciences and Health (UM6SS), Casablanca, MAR; 6 Medical Oncology, Cheikh Khalifa International University Hospital, Casablanca, MAR

**Keywords:** bone marrow-sparing, clinical outcomes, dose-volume parameters, hematologic toxicity, pelvic radiotherapy, radiation-induced lymphopenia

## Abstract

Pelvic radiotherapy is widely used in the treatment of several common malignancies but is frequently accompanied by hematologic toxicity due to incidental irradiation of active bone marrow within the pelvis. Growing interest has therefore focused on strategies aimed at reducing unnecessary marrow exposure while preserving target coverage and treatment effectiveness. This narrative review provides an overview of the biological basis of radiation-induced marrow injury, current approaches to bone marrow identification and sparing, and the clinical relevance of these strategies across major pelvic cancer sites. Available evidence suggests that limiting bone marrow irradiation can reduce treatment-related hematologic toxicity, particularly in patients receiving combined modality therapy, and may have implications for immune preservation. However, the extent to which marrow-sparing techniques influence long-term oncologic outcomes remains uncertain. Further prospective research is required to refine planning strategies and to better define the clinical role of bone marrow preservation in pelvic radiotherapy.

## Introduction and background

Pelvic radiotherapy plays a central role in the management of several common malignancies, including rectal, cervical, endometrial, and prostate cancers. It is used in multiple clinical settings, definitive, neoadjuvant, adjuvant, and palliative, and is often combined with systemic treatments [1-4]. Despite major technological advances that have improved dose conformity and reduced exposure to critical organs, hematologic toxicity remains a frequent and clinically relevant consequence of pelvic irradiation [5].

In adults, the pelvic skeleton contains a substantial proportion of active hematopoietic bone marrow, accounting for approximately 40% of the total functional marrow reserve. This tissue is highly radiosensitive, and even relatively low radiation doses can impair hematopoiesis by damaging proliferating progenitor cells and the supporting stromal environment [6]. Consequently, pelvic radiotherapy is commonly associated with declines in circulating blood cell counts, including leukopenia and lymphopenia, which may negatively affect immune function, treatment tolerance, and clinical outcomes [7].

Hematologic toxicity is more pronounced when pelvic radiotherapy is delivered concurrently with chemotherapy, as commonly practiced in rectal and cervical cancer [5]. Multiple studies have demonstrated a consistent association between radiation dose to pelvic bone marrow and the risk of clinically significant cytopenias. Parameters such as mean bone marrow dose and low-dose exposure metrics, including V5 to V20, have been repeatedly identified as predictors of grade ≥2 hematologic toxicity [8].

Beyond acute toxicity, radiation-induced lymphopenia has emerged as a potential prognostic factor. Lymphocytes are among the most radiosensitive cells in the body, and their depletion during radiotherapy has been associated with poorer local control and survival in several solid tumors. These findings suggest that bone marrow irradiation may influence outcomes not only through hematologic toxicity but also through effects on antitumor immune responses [9].

The widespread use of intensity-modulated radiotherapy, volumetric modulated arc therapy, and image-guided techniques has made bone marrow-sparing strategies feasible in routine clinical practice. Several studies have reported reduced hematologic toxicity when bone marrow constraints are incorporated into treatment planning, without compromising target coverage [10]. However, important uncertainties remain regarding optimal bone marrow definition, dosimetric thresholds, and the clinical significance of marrow sparing for long-term oncologic outcomes [9].

Against this background, a comprehensive and clinically focused review is warranted. This review summarizes the anatomical and functional characteristics of pelvic bone marrow, examines dosimetric parameters associated with hematologic toxicity, and reviews clinical outcomes related to bone marrow-sparing pelvic radiotherapy across major pelvic malignancies. The aim is to clarify the clinical relevance of bone marrow preservation and to highlight future directions for optimizing pelvic radiotherapy in an era of increasing treatment individualization.

## Review

Scope of the review and literature selection

This article is presented as a narrative review aimed at providing a clinically oriented synthesis of biological, dosimetric, and outcome-related evidence on bone marrow-sparing pelvic radiotherapy. No formal systematic review or meta-analysis was intended. The literature discussed was identified through focused searches of PubMed and Scopus, supplemented by manual review of references from key articles. Study selection was based on clinical relevance and methodological quality, and findings were synthesized qualitatively.

Bone marrow anatomy and radiosensitivity

Bone marrow is a dynamic and spatially heterogeneous organ that plays a fundamental role in adult hematopoiesis. In mature individuals, active hematopoietic marrow is predominantly located within the axial skeleton, with the pelvic bones, including the ilium, sacrum, ischium, pubis, and lower lumbar vertebrae, containing the largest proportion of functional marrow reserves [6]. This anatomical distribution has direct clinical implications in pelvic radiotherapy, where substantial volumes of active marrow are inevitably included within treatment fields and exposed to a spectrum of radiation doses [5].

From a radiobiological standpoint, the bone marrow is characterized by a high degree of radiosensitivity, driven primarily by the intrinsic vulnerability of hematopoietic stem and progenitor cells and their reliance on an intact microenvironment for survival and regeneration. These progenitor populations exhibit a high proliferative activity and limited tolerance to DNA damage, making them particularly susceptible to radiation-induced double-strand breaks. As a result, even relatively low radiation doses can precipitate apoptosis, mitotic failure, or cellular senescence in clonogenic precursors, leading to early and clinically meaningful suppression of hematopoietic function [11].

Importantly, radiation-induced marrow injury is not confined to direct damage of hematopoietic cells but also involves disruption of the bone marrow niche. The stromal compartment, including mesenchymal stem cells, endothelial cells, osteoblasts, and fibroblasts, plays a pivotal role in maintaining hematopoietic stem cell homeostasis through complex cellular interactions and tightly regulated cytokine signaling. Radiation-related alterations of this microenvironment compromise vascular integrity and stromal support, thereby limiting hematopoietic recovery, even in regions exposed to sub-ablative radiation doses [12].

Bone marrow toxicity also demonstrates a pronounced volume-dependent effect. While high-dose irradiation results in localized marrow ablation, exposure of large marrow volumes to low and intermediate doses, commonly encountered in modern pelvic radiotherapy, can produce substantial cumulative hematopoietic suppression [13]. This phenomenon is further amplified during fractionated treatment, as circulating blood elements repeatedly traverse irradiated marrow-rich regions, contributing to sustained depletion of radiosensitive cell populations. Consequently, both dose intensity and irradiated volume are critical determinants of radiation-related hematologic toxicity [14].

Among hematologic manifestations, lymphopenia represents a particularly sensitive indicator of marrow radiosensitivity. Lymphocytes are among the most radiosensitive cells in the human body, and their depletion during radiotherapy reflects a combination of direct irradiation of circulating lymphocytes, impaired lymphopoiesis within the marrow, and altered immune cell trafficking secondary to stromal and vascular injury [15]. Together, these mechanisms provide a biologically coherent explanation for the association between pelvic bone marrow irradiation, lymphocyte depletion, and adverse oncologic outcomes, reinforcing the rationale for considering bone marrow as a critical organ at risk in contemporary pelvic radiotherapy planning [16].

Molecular and microenvironmental determinants

At the molecular level, bone marrow radiosensitivity reflects intrinsic properties of hematopoietic stem and progenitor cells and their reliance on tightly regulated niche signaling. Homeobox transcription factors, particularly HOXA9 and HOXB4, play a central role in stem cell self-renewal, proliferation, and lineage commitment, notably through regulation of cell-cycle progression and DNA damage responses. Disruption of HOX-dependent transcriptional programs following irradiation has been associated with impaired clonogenic survival and delayed hematopoietic recovery [17].

Post-transcriptional regulation also contributes to marrow radiosensitivity. miR-155 is a key modulator of hematopoietic and immune cell function and influences progenitor cell proliferation, survival, and responses to genotoxic stress. Perturbation of miR-155-dependent pathways after irradiation may therefore further compromise clonogenic potential and delay bone marrow recovery [18].

In parallel, Kruppel-like factors such as KLF1 and KLF4 modulate hematopoietic differentiation and cellular quiescence. Perturbation of KLF-mediated regulation following irradiation contributes to defective erythroid and lymphoid maturation, amplifying marrow suppression beyond the direct effects of DNA damage. Thus, progenitor radiosensitivity is driven not only by genotoxic injury but also by altered transcriptional control of stem cell fate [19].

Radiation exposure also compromises the integrity of the bone marrow microenvironment. Key niche signaling pathways, including the CXCL12-CXCR4 axis, which governs stem cell homing and retention, and stem cell factor (SCF)/c-KIT signaling, essential for progenitor survival, are particularly vulnerable to stromal and endothelial injury. Disruption of these pathways results in impaired stem cell anchorage, altered trafficking, and limited regenerative capacity [20].

Additionally, irradiation induces sustained microenvironmental changes characterized by endothelial dysfunction, reduced vascular perfusion, and increased TGF-β-mediated profibrotic signaling. These alterations promote stem cell exhaustion or prolonged quiescence and interfere with immune cell differentiation and migration. Importantly, such effects may persist beyond acute cytopenias, contributing to prolonged lymphopenia and immune dysregulation [21].

Taken together, bone marrow radiosensitivity emerges from a complex interplay between intrinsic stem cell vulnerability, transcriptional dysregulation, and niche disruption. This integrated biological framework provides mechanistic support for the clinical association between marrow irradiation, lymphocyte depletion, and adverse oncologic outcomes, and reinforces the rationale for bone marrow-sparing strategies in pelvic radiotherapy.

Dosimetric considerations and bone marrow contouring

Accurate delineation of pelvic bone marrow is a prerequisite for meaningful dosimetric optimization. In routine clinical practice, bone marrow is most commonly contoured using the entire pelvic bony anatomy on planning CT scans, including the ilium, sacrum, ischium, pubis, and lower lumbar vertebrae [5]. This anatomical approach is simple, reproducible, and widely applicable, although it does not distinguish between active and inactive marrow compartments [5]. Functional imaging-guided contouring, particularly using PET-based techniques, has been explored to better identify metabolically active marrow subvolumes; however, heterogeneity in methodology, availability, and validation currently limits its routine clinical implementation [6]. Alternative contouring strategies based on functional or advanced anatomical imaging have been explored. In particular, MRI-based approaches have been shown to improve identification of active bone marrow compared with CT alone, with studies in cervical cancer demonstrating differences in marrow delineation and potential implications for bone marrow-sparing [22].

From a dosimetric perspective, pelvic bone marrow behaves as a parallel organ with a pronounced volume-dependent response to irradiation [14]. Clinical studies consistently demonstrate that hematologic toxicity is influenced not only by high-dose exposure but also by the cumulative irradiation of large marrow volumes to low and intermediate doses. Accordingly, dosimetric parameters such as mean bone marrow dose and low-dose volume metrics have emerged as relevant predictors of treatment-related cytopenias [5].

Although no universally accepted dose constraints exist, several retrospective analyses, including those by Mell et al., Chang et al., and Brixey et al., as well as a prospective phase II study by Williamson et al., have proposed dose-volume parameters associated with reduced hematologic toxicity [5,23-25].

The most consistently reported metrics emphasize limiting low- and intermediate-dose exposure, with commonly cited objectives including a mean bone marrow dose of approximately ≤30 Gy, V10 below 90-95%, V20 below 70-75%, and V30 below 50-60%, when feasible [5]. These dose-volume parameters are primarily intended to reduce hematologic toxicity and preserve treatment tolerance. In the setting of concurrent systemic therapy, myelosuppression is driven by multiple interacting factors, which substantially limit the ability to isolate the independent impact of radiotherapy dose-volume parameters on overall survival.

Modern radiotherapy techniques, such as intensity-modulated radiotherapy and volumetric modulated arc therapy, enable the incorporation of bone marrow as an organ at risk during treatment planning without compromising target coverage. Rather than adhering to rigid dose thresholds, contemporary bone marrow-sparing strategies emphasize global reduction of unnecessary low- and intermediate-dose exposure while maintaining oncologic adequacy and respecting competing organ-at-risk constraints. This individualized approach is particularly relevant in patients at increased risk of hematologic toxicity or prolonged lymphopenia [23]. An overview of the principal clinical studies examining pelvic bone marrow dose-volume parameters and hematologic toxicity in pelvic radiotherapy is provided in Table 1.

**Table 1:** Clinical studies assessing pelvic bone marrow irradiation and hematologic toxicity in pelvic malignancies IMRT: intensity-modulated radiotherapy; 3D-CRT: three-dimensional conformal radiotherapy; VMAT: volumetric modulated arc therapy; WPRT: whole pelvic radiotherapy; IM-WPRT: intensity-modulated whole pelvic radiotherapy; BM: bone marrow; BMTOT: total pelvic bone marrow; BMLS: lumbosacral bone marrow; LSS: lumbosacral spine; Gy: gray

Study	Cancer site/population	Radiotherapy technique	Bone marrow definition	Dosimetric parameters evaluated	Main findings
Mell et al. (2006) [5]	Cervical cancer (concurrent chemoradiotherapy)	IMRT	Whole pelvic bony anatomy contoured on planning CT	Mean bone marrow dose, V10, V20	Higher mean dose and increased low-dose volumes were associated with higher rates of acute hematologic toxicity
Chang et al. (2016) [23]	Cervical cancer patients (chemoradiotherapy)	IMRT vs 3D-CRT vs RapidArc	Pelvic bone marrow contoured on CT	Bone marrow dose–volume parameters	IMRT associated with milder hematologic toxicity compared with 3D-CRT and RapidArc; dosimetric parameters correlated with incidence/severity of hematologic toxicity
Brixey et al. (2002) [24]	Women with gynecologic malignancies (cervix, uterus)	IM-WPRT vs conventional WPRT	Pelvic bone marrow contoured anatomically on CT	Bone marrow dose–volume histograms	IM-WPRT resulted in reduced pelvic marrow dose and lower rates of some acute hematologic toxicities compared with conventional radiotherapy
						Hui et al. (2014) [26]	Cervical cancer patients (concurrent chemoradiotherapy)	IMRT vs 3D-CRT	Pelvic bones contoured anatomically on CT	V10, V20, V30, V40, V50	IMRT reduced high-dose pelvic marrow volumes (V30–V50) compared with 3D-CRT, and acute hematologic toxicity was more frequent/severe in the 3D-CRT group than in the IMRT group
Wan et al. (2015) [27]	Rectal cancer patients (concurrent chemoradiotherapy)	IMRT	Pelvic bone marrow contoured anatomically on CT	Lumbosacral spine V40	Increased lumbosacral spine V40 was significantly associated with grade ≥2 acute hematologic toxicity; maintaining LSS-V40 <60% reduced the risk of grade ≥2 toxicity compared with higher V40 values (p < 0.05)						
												Pavarini et al. (2024) [28]	Prostate cancer with pelvic node irradiation	IMRT	Pelvic bone marrow contoured anatomically on CT (ilium, lumbosacral, lower pelvis & whole pelvis)	BMLS-V24Gy, BMTOT-V10Gy, V20Gy, V30Gy	Late grade ≥2 lymphopenia was independently associated with baseline ALC and BMLS-V24; suggested constraints (BMTOT V10 <1520 cc, V20 <1250 cc, V30 <850 cc, and BMLS-V24 <307 cc) to potentially reduce risk of late lymphopenia

Clinical evidence by tumor site

Cervical Cancer

In cervical cancer, the relationship between pelvic bone marrow irradiation and hematologic toxicity has been the most extensively investigated, largely due to the routine use of concurrent chemoradiotherapy. Multiple clinical studies consistently demonstrate a strong association between bone marrow dose-volume parameters and the incidence and severity of acute hematologic toxicity [5,10,25,27]. Early work using intensity-modulated radiotherapy identified mean pelvic bone marrow dose and low-dose volume metrics (V10-V20) as significant predictors of treatment-related cytopenias [5,29]. Subsequent comparative studies have shown that, compared with three-dimensional conformal radiotherapy, IMRT and VMAT reduce the integral dose to pelvic bone marrow by limiting exposure to intermediate- and high-dose regions, which is associated with lower rates of acute hematologic toxicity. More recent data further support these findings, confirming that dose-volume parameters across a broad dose range correlate with both the incidence and severity of hematologic toxicity in patients undergoing concurrent chemoradiotherapy [10]. Collectively, the cervical cancer literature provides robust clinical evidence supporting the integration of bone marrow-sparing objectives into pelvic radiotherapy planning, particularly in patients receiving myelosuppressive systemic therapy.

Rectal Cancer

In rectal cancer, clinical evidence linking bone marrow irradiation to hematologic toxicity remains more limited than in gynecologic malignancies and is subject to several important sources of heterogeneity. First, the increasing use of intensified systemic therapy within total neoadjuvant treatment (TNT) strategies introduces variability in chemotherapy exposure, which substantially influences hematologic toxicity independently of radiotherapy [30,31]. Second, differences in radiotherapy prescription, including the use of short-course regimens (5 × 5 Gy) [31] versus conventionally fractionated long-course chemoradiotherapy (approximately 50 Gy in 25 fractions) [30], result in distinct dose distributions and patterns of bone marrow exposure.

In contrast to cervical cancer, where large pelvic fields are routinely treated, rectal cancer target volumes are anatomically closer to the sacrum and lumbosacral spine, leading to preferential irradiation of this marrow subregion. Clinical studies have therefore identified the lumbosacral spine as a particularly relevant component of pelvic bone marrow, with higher intermediate-dose exposure associated with an increased risk of acute hematologic toxicity [8,27,32]. Although data remain limited, available evidence, including dosimetric and clinical studies using VMAT/RapidArc techniques, suggests that selective sparing of lumbosacral marrow, when technically feasible, may reduce hematologic toxicity without compromising target coverage in rectal cancer patients receiving chemoradiotherapy [33].

Prostate Cancer

In prostate cancer, the clinical implications of pelvic bone marrow irradiation differ substantially from those observed in rectal and gynecologic malignancies. Concurrent chemotherapy is uncommon, and hematologic toxicity most frequently manifests as treatment-related lymphopenia rather than multilineage cytopenias. Clinical studies evaluating pelvic radiotherapy in prostate cancer, particularly in patients undergoing pelvic nodal irradiation, have demonstrated a clear association between bone marrow dose-volume parameters and both acute and late lymphopenia [28,34]. Higher mean pelvic bone marrow dose and increased low- and intermediate-dose exposure have been linked to greater lymphocyte depletion, with recent data identifying specific dose-volume metrics of lumbosacral and total pelvic marrow as independent predictors of late grade ≥2 lymphopenia [28]. These findings underscore the immunological relevance of bone marrow sparing in prostate cancer, especially in patients receiving extended-field radiotherapy, and support the consideration of marrow preservation as part of individualized treatment planning [35].

Overall, the strength of evidence supporting bone marrow-sparing strategies is highest in cervical cancer, moderate in rectal cancer, and more limited in prostate cancer, reflecting differences in treatment paradigms, use of concurrent chemotherapy, and available study designs.

Clinical outcomes

Beyond acute hematologic toxicity, several clinical studies have investigated the relationship between lymphocyte dynamics, treatment response, and longer-term outcomes in patients undergoing pelvic radiotherapy for rectal and prostate cancers.

In rectal cancer, evidence linking host immune status to tumor response has been reported. Kitayama et al. found that higher circulating lymphocyte counts during neoadjuvant chemoradiotherapy were positively associated with tumor response in patients with advanced rectal cancer, suggesting that preservation of lymphocyte levels may correlate with better pathologic outcomes following CRT [36]. Additionally, Wu et al. demonstrated that decreases in circulating lymphocyte count during neoadjuvant therapy were associated with improved tumor regression, indicating a complex relationship between lymphocyte dynamics and tumor response in rectal cancer [37].

Regarding the effects of bone marrow-sparing radiotherapy on clinical outcomes, a study by Huang et al. on pelvic bone marrow-sparing IMRT in rectal cancer patients receiving concurrent capecitabine-based chemoradiotherapy showed that application of specific bone marrow dose constraints (V10 ≤ 85%, V20 ≤ 65%, V30 ≤ 45%) significantly reduced the incidence of grade 3 acute hematologic toxicity. Although this study did not directly assess long-term survival endpoints, the reduced hematologic toxicity observed with bone marrow sparing has potential implications for treatment delivery and overall outcomes by facilitating uninterrupted chemoradiotherapy [32].

In cervical cancer, the clinical relevance of treatment-related lymphopenia has been specifically highlighted by Taguchi et al., who reported that persistent lymphopenia following radiotherapy was a significant adverse prognostic factor in patients treated with definitive pelvic radiotherapy. In this single-center retrospective study, patients who developed sustained post-treatment lymphopenia experienced inferior survival outcomes compared with those who maintained higher lymphocyte counts, underscoring the clinical importance of immune preservation in this context. Although this study did not directly correlate lymphocyte depletion with bone marrow dose-volume parameters, its findings are biologically and clinically consistent with the extensive irradiation of pelvic bone marrow inherent to cervical cancer radiotherapy [38].

Across pelvic malignancies, treatment-related lymphopenia has been consistently associated with inferior clinical outcomes, including reduced tumor response and survival. Although a direct survival benefit of bone marrow-sparing strategies has not yet been conclusively demonstrated, the recurrent association between pelvic bone marrow irradiation, lymphocyte depletion, and adverse outcomes supports the clinical relevance of minimizing unnecessary marrow exposure during pelvic radiotherapy [9].

Future directions

Future research in bone marrow-sparing pelvic radiotherapy should focus on prospective validation of dosimetric strategies using standardized delivery techniques and contouring methods. Studies should incorporate consistent definitions of pelvic bone marrow, predefined dose-volume objectives, and systematic hematologic assessment to improve comparability across institutions and enable the development of reproducible constraints.

Further work is needed to determine whether functional imaging-based bone marrow delineation offers clinically relevant advantages over anatomical contouring in routine practice, particularly in the context of modern IMRT and VMAT planning. In addition to acute hematologic toxicity, future studies should assess longer-term immune effects and oncologic endpoints, including tumor control and survival, recognizing the confounding impact of systemic therapy in most pelvic malignancies.

In prostate cancer, the relevance of highly conformal approaches such as stereotactic body radiotherapy (SBRT) warrants specific evaluation. Given the smaller treatment volumes and abbreviated fractionation schedules, SBRT may inherently limit bone marrow exposure, potentially reducing the clinical importance of marrow-sparing constraints. This hypothesis requires confirmation in dedicated clinical and dosimetric studies, particularly in patients receiving pelvic nodal irradiation.

## Conclusions

Taken together, the available evidence indicates that excessive irradiation of pelvic bone marrow contributes to treatment-related hematologic toxicity and lymphopenia, which may, in turn, influence clinical outcomes in patients receiving pelvic radiotherapy. Clinical and dosimetric studies across pelvic malignancies suggest that limiting unnecessary bone marrow exposure, particularly to low and intermediate radiation doses, can reduce hematologic toxicity without compromising target coverage. Although direct evidence linking bone marrow-sparing strategies to improved survival remains limited, observed associations between marrow dose-volume parameters, lymphocyte depletion, and adverse outcomes support continued integration of bone marrow as an organ at risk in pelvic radiotherapy planning. Prospective studies are needed to define optimal dose constraints and to determine whether bone marrow-sparing pelvic radiotherapy can translate into meaningful improvements in tumor control and patient outcomes.
